# Generic calibration of a simple model of diurnal temperature variations for spatial analysis of accumulated degree-days

**DOI:** 10.1007/s00484-017-1471-5

**Published:** 2017-12-07

**Authors:** Raphael Felber, Sibylle Stoeckli, Pierluigi Calanca

**Affiliations:** 1 Agroscope, Research Division Agroecology and Environment, Climate and Air Pollution Group, Zurich, Switzerland; 2 Department of Crop Sciences, Research Institute of Organic Agriculture (FiBL), Frick, Switzerland

**Keywords:** Hourly temperature model, Accumulated growing degree-days, Phenological dates, Spatial variation

## Abstract

Accumulated growing degree-days (aGDD) are widely used to predict phenological stages of plants and insects. It has been shown in the past that the best predictive performance is obtained when aGDD are computed from hourly temperature data. As the latter are not always available, models of diurnal temperature changes are often employed to retrieve the required information from data of daily minimum and maximum temperatures. In this study, we examine the performance of a well-known model of hourly temperature variations in the context of a spatial assessment of aGDD. Specifically, we examine whether a generic calibration of such a temperature model is sufficient to infer in a reliable way spatial patterns of key phenological stages across the complex territory of Switzerland. Temperature data of a relatively small number of meteorological stations is used to obtain a generic model parameterization, which is first compared with site-specific calibrations. We show that, at the local scale, the predictive skill of the generic model does not significantly differ from that of the site-specific models. We then show that for aGDD up to 800 °C d (on a base temperature of 10 °C), phenological dates predicted with aGDD obtained from estimated hourly temperature data are within ± 3 days of dates estimated on the basis of observed hourly temperatures. This suggests the generic calibration of hourly temperature models is indeed a valid approach for pre-processing temperature data in regional studies of insect and plant phenology.

**Electronic supplementary material:**

The online version of this article (10.1007/s00484-017-1471-5) contains supplementary material, which is available to authorized users.

## Introduction

Air temperature is the main determinant of plant and insect growth (Huey and Stevenson 1979; Deutsch et al. 2008), a well-known fact that led to the development of conceptual models relating plant and insect phenology to temperature (as a measure of heat availability) already in the middle of the eighteenth century (Allen 1976; Wilson and Barnett 1983). Particularly important in this context is the total amount of heat required for an organism to develop from one point to another in its life cycle (Baskerville and Emin 1969). This is usually expressed in terms of accumulated growing degree-days (aGDD), that is to say, the integral over a given period of time of the daily excess of temperature over a lower developmental threshold, the so-called base temperature (Baskerville and Emin 1969; Prentice et al. 1992).

The degree-day approach assumes a linear relationship between development rate and temperature (Riedl 1983; Roltsch et al. 1999; Snyder et al. 1999). It requires specification of organism dependent temperature thresholds that can be derived from laboratory experiments (Pitcairn et al. 1991) or field observations (Snyder et al. 1999). The approach has been extensively used in agricultural fields to predict harvest times, schedule planting dates of crops, or to plan disease, weed, and pest control applications (e.g. Worner 1988). In recent years, the approach has also been adopted for climate change impact assessments (e.g. Grigorieva et al. 2010; Stoeckli et al. 2012; Juszczak et al. 2013; Bethere et al. 2016).

Comparison of degree-day estimates obtained from daily and hourly data has shown that the latter should be preferred, whenever possible (Worner 1988; Reicosky et al. 1989; Roltsch et al. 1999; Cesaraccio et al. 2001; Purcell 2003; Gu 2016). Unfortunately, hourly data is not always available. For this reason, models for simulating diurnal temperatures variations from daily minimum (*T*_n_) and maximum temperature (*T*_x_) have been proposed in the past (Parton and Logan 1981; Eckersten 1986; Worner 1988; Linvill 1990; Tejeda Martinez 1991; Cesaraccio et al. 2001; Chow and Levermore 2007; Eccel 2010a; Horton 2012; Kearney et al. 2014). Irrespective of the specific choice, all models assume that observed temperature variations follow regular diurnal temperature patterns. This usually is the case for clear-sky conditions over flat terrain, but less so on overcast or rainy days (Reicosky et al. 1989) or in complex terrain.

Models of diurnal temperature variations are expected to also play an important role for climate change impact assessments. In fact, while it is true that current global or regional climate models do compute temperature (and other variables) at high temporal and spatial resolution, the possibility to use model outputs directly for further analysis is ruled out by the presence of systematic errors, and the relatively course spatial resolution of the climate models. Downscaling and bias correction techniques are used for the post-processing of climate model output and the development of reliable regional climate change scenarios (Wilby et al. 2009; Calanca and Semenov 2013). Yet, these procedures typically aim at the daily timescale.

Increasing computational power, the availability of weather records at sub-daily scale and of satellite imagery have made it possible to use more sophisticated schemes to estimate degree-days (Floyd and Braddock 1984; Reicosky et al. 1989; Kean 2013) and to apply degree-day models at the spatial scale (e.g. Hassan et al. 2007; Kean 2013; Spinoni et al. 2015; Wypych et al. 2017). For an overview of different approaches for the calculation of degree-days, see Zalom et al. (1983), Cesaraccio et al. (2001), and Rodríguez Caicedo et al. (2012) and references therein.

In this study, we investigate the potential for using a simple, widely used model of diurnal temperature variations (Parton and Logan 1981) in spatial analysis of accumulated degree-days in Switzerland, a country characterised by complex topography and a wide range of local thermal regimes. The model assumes a sinus function and an exponential decay to simulate day-time and night-time temperatures, respectively. In addition to ${T}_{\mathrm{n}}$ and ${T}_{\mathrm{x}}$ as well as sunrise and sunset hours as input data, the model involves only three parameters that are easily calibrated (e.g. Parton and Logan 1981; Eckersten 1986). It has been shown earlier that the range of model parameter values across sites tends to be narrow, suggesting that in many circumstances even a generic calibration can deliver satisfactory results (Reicosky et al. 1989). A specific goal of our study was to develop a generic model for the whole of Switzerland using hourly temperature data from only ten meteorological stations and test its performance for computing accumulated degree-days in comparison to specific models obtained from individual parameterizations.

## Material and methods

### The model

For this work, we adopted the model of Parton and Logan (1981) but with improvements concerning (i) the phase shift of the sinusoid invoked to simulate day-time temperature variations (the curve was forced to run through maximum temperature), (ii) the exponential decay at night (an additive term was included to force the curve through minimum temperature), and (iii) the specification of temperature at sunset and minimum temperature to model the exponential decay in the early morning hours and the late evening hours (information from the previous and next day was included as appropriate).

With this, the following set of equations (Eq. 1) describes the improved temperature model:

$$ T{(h)}_i=\left\{\begin{array}{lll}{T}_{\mathrm{n},i}+\left({T}_{\mathrm{S},i-1}-{T}_{\mathrm{n},i}\right){e}^{\frac{-b\left(h-{h}_{\mathrm{S},i-1}+24\right)}{n_1}}-\frac{h-{h}_{\mathrm{S},i-1}+24}{n_1}{e}^{-b}& \mathrm{for}\ h<{h}_{\mathrm{R},i}+c& \mathrm{Eq}.\left(1\mathrm{a}\right)\\ {}{T}_{\mathrm{n},i}+\left({T}_{\mathrm{x},i}-{T}_{\mathrm{n},i}\right)\sin \left(\frac{\uppi \left(h-{h}_{\mathrm{R},i}-c\right)}{h_{\mathrm{S},i}-{h}_{\mathrm{R},i}+2a-2c}\right)& \mathrm{for}\ h\ge {h}_{\mathrm{R},i}+c\ \mathrm{and}\ h\le {h}_{\mathrm{S},i}& \mathrm{Eq}.\left(1\mathrm{b}\right)\\ {}{T}_{\mathrm{n},i+1}+\left({T}_{\mathrm{S},i}-{T}_{\mathrm{n},i+1}\right){e}^{\frac{-b\left(h-{h}_{\mathrm{S},i}\right)}{n_2}}-\frac{h-{h}_{\mathrm{S},i}}{n_2}{e}^{-b}& \mathrm{for}\ h>{h}_{\mathrm{S},i}& \mathrm{Eq}.\left(1\mathrm{c}\right)\end{array}\right. $$

where *T*(*h*) is the temperature at hour (*h*) of day *i*, ${T}_{\mathrm{n}}$ and ${T}_{\mathrm{x}}$ are the daily minimum and maximum temperature; ${T}_{\mathrm{S}}$ is the sunset temperature (calculated with Eq. 1b for ${h}_{\mathrm{S}}$); ${n}_{\mathsf{1}}$ the corrected night length before sunrise (${n}_1={h}_{\mathrm{R},i}\kern.3em -\kern.3em {h}_{\mathrm{S},i-1}+c+24$) and ${n}_{\mathsf{2}}$ the corrected night length after sunset until the sunrise of the following day (${n}_2={h}_{\mathrm{R},i+1}-{h}_{\mathrm{S},i}+c+24$); ${h}_{\mathrm{R}}$ and ${h}_{\mathrm{S}}$ are sunrise and sunset hours, respectively, and *a* is the lag coefficient for ${T}_{\mathrm{x}}$ from noon, *b* the night-time temperature decay coefficient and *c* the time lag for ${T}_{\mathrm{n}}$ from sunrise.

Sunrise (${h}_{\mathrm{R}}$) and sunset hour (${h}_{\mathrm{S}}$) were calculated for each site as a function of geographic latitude and day of the year (Iqbal 1983).

### Sites and temperature data

**Fig. 1:**
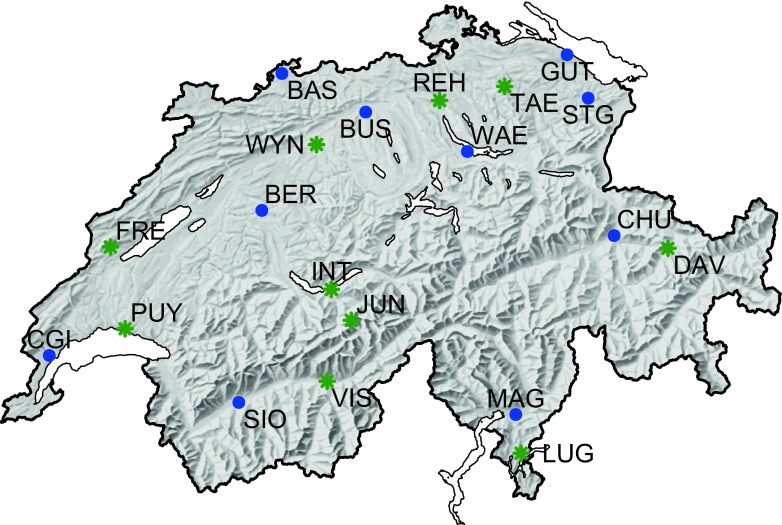
Locations of the 20 meteorological stations in Switzerland used in this study. Blue dots indicate sites used for model calibration and validation (years 1981–2015). Green stars indicate sites used for assessment of the generic model (years 1988–2015)

Hourly air temperature data measured at 2-m height above ground from 20 meteorological stations in Switzerland (Fig. 1) as provided by the Federal Office of Meteorology and Climatology (MeteoSwiss 2016) were used in this study. Full names, coordinates and data availability are listed in Table S1 (Supplementary Material). Most of these sites are located in agricultural areas, at elevations below 800 m.a.s.l. Two of them, FRE and DAV (Bullet/La Frêtaz and Davos), are located above 1100 m.a.s.l. They were included to test the model performance in high-elevation agricultural areas. Finally, JUN (Jungfraujoch) is located at 3580 m.a.s.l. It is a high alpine station with no relevance for agriculture. The site was nevertheless taken into account to test the suitability of the hourly temperature model under extreme conditions.

The model operates with true solar time (TST), but the hourly temperature data is given in mean local time. To synchronise the data, mean local time was converted into TST on the basis of Eq. (1.4.1) and Eq. (1.4.2) in Iqbal (1983).

For each site, daily minimum and maximum temperatures were obtained from the hourly data. Ten sites (BAS, BER, BUS, CGI, CHU, GUT, MAG, SIO, STG and WAE; blue points in Fig. 1) denoted as ‘calibration sites’, were used for developing the generic model. The other ten sites (DAV, FRE, INT, JUN, LUG, PUY, REH, TAE, VIS and WYN; green stars in Fig. 1) were used to assess the potential for spatial application of the generic model. These sites are referred as ‘validation sites’ in the following.

### Model calibration and validation

The model was calibrated for each site individually, giving 20 site-specific parameterizations. We refer to this set as the ‘site-specific models’. Additionally, a single calibration was carried out for the ten calibration sites taken together. In the following, this will be referred to as the ‘generic model’. Twenty-five randomly selected years were used for the calibration.

Following Reicosky et al. (1989), only ‘clear-sky’ days were considered for the calibration. They were selected on the basis of ${T}_{\mathrm{n}}$ occurring before noon and the ratio between observed and potential solar radiation, assuming for the latter a threshold of 0.9.

Parameter fitting was carried out with the ‘Nelder-Mead’ method, as implemented in the function ‘optim’ of the R software (Version 3.2.2, R Core Team 2016). The modified index of agreement (MIA; Legates and McCabe 1999) was used as a performance metric for the optimization.

To assess the model performance, the following metrics were used: mean error (ME), mean absolute error (MAE), root mean square deviation (RMSD), modified index of agreement (MIA), Nash-Sutcliffe efficiency (NSE) and coefficient of determination (R^2^). The performance statistics were evaluated using the R library ‘hydroGOF’ (Zambrano-Bigiarini 2012).

### Accumulated growing degree-days

Accumulated growing degree-days, which we denote as aGDD (°C d) in accordance with the terminology introduced in the Glossary of Biometeorology (Gosling et al. 2014), were computed from (observed or simulated) hourly temperatures as (Purcell 2003):

2 $$ \mathrm{aGDD}(k)=\frac{1}{24}{\sum}_{d=1}^k{\sum}_{h=1}^{24}\max \left(0,{T}_h(d)-{T}_{\mathrm{b}}\right) $$

where *k* is the upper summation limit (day of the year or DOY), ${T}_h$ the air temperature of the *h*-th hour of day (*d*) and ${T}_{\mathrm{b}}$ the base temperature. In our examples, we set *T*_b_ = 10 °C, which is roughly in the middle of the range of base temperatures adopted for modelling insect phenology (Pruess 1983) but at the upper end of the range of base temperatures that apply to insects found in Mid-Europe. As shown by Pruess (1983), the performance of models estimating aGDD from diurnal temperature variations degrades with increasing base temperature. We hence consider the choice of an elevated *T*_b_ as pertinent for addressing model performance.

The distribution of DOYs corresponding to a given aGDD value was examined by means of the Wilcoxon-Mann-Whitney test and the Kolmogorov-Smirnoff test, using the respective R functions (R Core Team 2016). In addition, a model efficiency (Ef) was defined in the spirit of Worner (1988) as the percentage of years and sites for which the estimated aGDD occurred within a ± 3-day window of the actual aGDD.

## Results

### Calibration and verification of the hourly temperature model

Table 1 compares the mean of model parameters (*a*, *b* and *c*) derived from the site-specific model calibration (site-specific temperature models) to the parameter values of the generic temperature model (more detailed information can be found in Table S2, Supplementary Material). Parameter values of the generic model lie within the range (mean ± 1 SD) of parameter values of the site-specific models. With respect to the site-specific models, note also that parameters *a* and *b* show lower relative variations than parameter *c*. Ancillary site-specific calibration of the hourly temperature model for the ten validation sites (Table S2) confirm these findings, except for the fact that at JUN and PUY, the values obtained for *a* are larger than 5.5, implying that in some cases the calibration procedure fails to provide realistic timing of ${T}_{\mathrm{x}}$.

**Table 1:** Mean and standard deviation (SD) of the site-specific model parameters *a*, *b* and *c* (upper line) and generic model parameters (lower line)

	Parameters		
	*a*	*b*	*c*
Site-specific	2.79 (0.29)	3.16 (0.36)	0.79 (0.27)
Generic	2.71	3.14	0.75

In principle, the model improvements implemented in Eq. 1 ensure that ${T}_{\mathrm{n}}$ and ${T}_{\mathrm{x}}$ are more accurately simulated than with the original model formulation. In practice, simulated ${T}_{\mathrm{n}}$ and ${T}_{\mathrm{x}}$ can still depart somewhat from the observed values because the phase shift parameters *a* and *c* are not necessarily multiples of the hours at which temperatures are simulated. Comparison of simulated ${T}_{\mathrm{n}}$ and ${T}_{\mathrm{x}}$ with observed ${T}_{\mathrm{n}}$ and ${T}_{\mathrm{x}}$ yields R^2^ larger than 0.98 and 0.99 for ${T}_{\mathrm{n}}$ and ${T}_{\mathrm{x}}$, respectively, with the site-specific model. The good performance of the site-specific models is further stressed by the statistics presented in Table S3 (Supplementary Material). For all sites, the Nash-Sutcliff efficiency (NSE) is larger than 0.94, and the MIA is larger than 0.91.

**Fig. 2:**
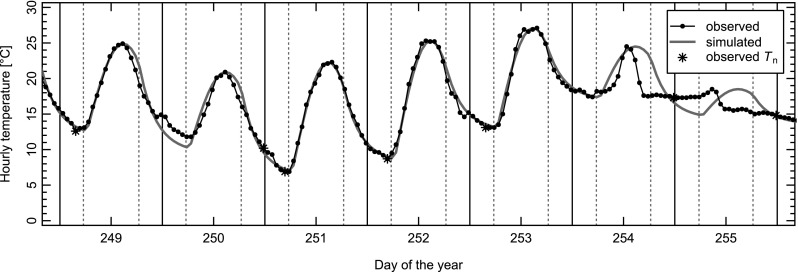
Temperature evolution at BAS during the summer of 1991. Dots and black line—observed temperatures. Grey line—simulated temperatures. The asterisks denote the minimum temperatures (${T}_{\mathrm{n}}$) extracted for each day from the corresponding 24 hourly values. Vertical solid lines indicate midnight, dashed vertical lines sunrise and sunset, respectively. Days 251 and 252 are classified as ‘clear-sky’ days (for definition see text)

Figure 2 shows observed and simulated (site-specific model) temperature variations during one week of the summer of 1991 at BAS, the site for which the model performance is best (MAE = 0.91, cf. Table S3). The figure verifies that the improved version of Parton and Logan’s (1981) model simulates well the transitions from one day to the next, which was not necessarily the case with the original formulation.

However, the figure also highlights four types of error that cannot be addressed during calibration:

i) Slight overestimation of observed temperatures in the late afternoon (DOY 249 to 253)
ii) Underestimation in the early morning, between midnight and sunrise (DOY 249 to 253)
iii) Failure of the assumed functional relations to describe the diurnal temperature course on overcast or rainy days (DOY 254 and 255)
iv) Error arising from a wrong attribution of ${T}_{\mathrm{n}}$ and ${T}_{\mathrm{x}}$ to fixed hours relatively to sunrise and sunset (DOY 250 and 255)

**Fig. 3:**
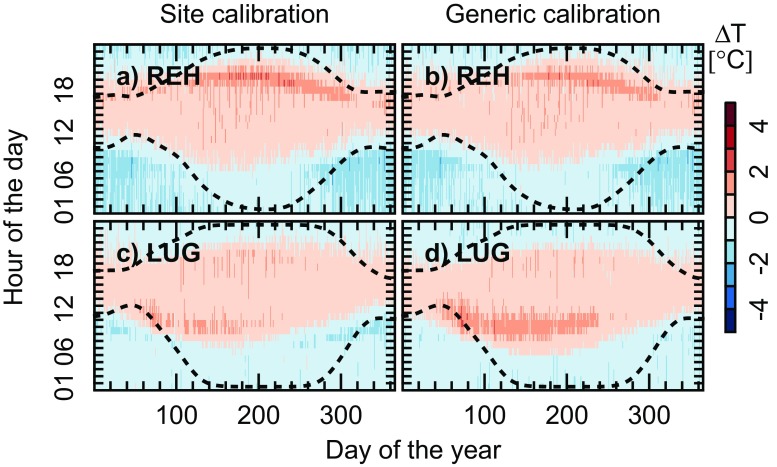
Mean for 1981–2015 of the difference between simulated and observed temperatures as a function of the time of the day (y-axis) and day of the year (x-axis), at REH (upper row) and LUG (lower row). Panels on the left present the mean deviations obtained with the site-specific models, whereas panels on the right show the deviations resulting from the application of the generic model. Reddish/blueish colours indicate a positive/negative bias. The dotted lines enclose the time of the day when *T* is in excess of *T*_b_ = 10 °C

Errors of types iii and iv tend to be larger than those of types i and ii, but the latter are responsible for the seasonal diurnal patterns of the difference between simulated and observed hourly data (Fig. S1, Supplementary Material). For example, for BAS (MAE = 0.91) and GUT (MAE = 1.06), differences are positive around midday and sunset, but negative between 5:00 and 6:00 and 16:00 and 18:00. This conclusion holds true irrespective of whether only ‘clear-sky’ or all days are considered and also irrespective of whether the specific or generic parameter values are used (Fig. 3 and Fig. S1). With the specific models for BAS and GUT, mean hourly deviation of clear-sky days range from − 4.56 to 3.31 °C and from − 6.79 to 4.70 °C when only ‘clear-sky’ cases are considered and from − 2.19 to 2.11 °C and − 2.45 to 2.95 °C when all days are included. Mean deviations for the specific models range from − 2.44 to 2.56 °C for REH and from − 1.87 to 2.16 °C for LUG. With the generic model, these ranges slightly increase: the values for REH vary between − 2.51 and 2.14 °C, while they vary between − 1.50 and 2.68 °C for LUG. As REH and LUG were not used for the calibration of the generic temperature model, the panels on the right can be considered as presenting an independent test of the generic model.

Table 2 presents a summary of the performance of the generic models at calibration and validation sites, stratified by time of the day and season. Analogous statistics for individual sites can be found in Table S4 (Supplementary Material). By and large, the statistics confirm that the generic model does perform less well than the site-specific models. However, because the site-specific models are calibrated for clear-sky days only, there are also sites performing slightly better with the generic model than with the site-specific model (e.g. BER, BUS and GUT with lower ME, MAE and RMSD values). Also, there is no systematic difference between the performance of the generic model at the calibration and validation sites (Table S4, Supplementary Material). The ME is in all cases negative, − 0.05 (± 0.09) and − 0.06 (± 0.10) °C for the calibration and validation sites, respectively. The overall negative bias is induced by a most pronounced underestimation during night-time (Table 2). If only hours for which $T\ge {T}_{\mathrm{b}}$ are taken into account (inner domain bounded by the two dashed lines in Fig. 3), then the ME becomes 0.22 (± 0.12) °C.

**Table 2:** Performance statistics (ME, MAE, RMSD, MIA, R^2^ and NSE) of the *generic temperature model* for selected hours of the day (04:00, 23:00, 10:00, 13:00) and seasons (spring, summer, fall, winter)

	ME (°C)	MAE (°C)	RMSD (°C)	MIA	R^2^	NSE
All	− 0.05 (0.09)	1.01 (0.13)	1.52 (0.18)	0.92 (0.01)	0.96 (0.01)	0.96 (0.01)
04:00	− 0.81 (0.23)	0.90 (0.21)	1.56 (0.28)	0.92 (0.02)	0.96 (0.01)	0.94 (0.02)
23:00	− 0.69 (0.15)	1.08 (0.12)	1.58 (0.20)	0.91 (0.01)	0.96 (0.01)	0.95 (0.02)
10:00	0.22 (0.33)	0.98 (0.09)	1.29 (0.12)	0.93 (0.01)	0.98 (0.01)	0.97 (0.01)
13:00	0.58 (0.21)	0.66 (0.17)	1.14 (0.26)	0.95 (0.02)	0.99 (0.01)	0.98 (0.02)
Spring	− 0.01 (0.11)	0.98 (0.08)	1.48 (0.13)	0.90 (0.01)	0.94 (0.02)	0.94 (0.02)
Summer	0.25 (0.10)	0.96 (0.07)	1.45 (0.11)	0.88 (0.02)	0.92 (0.02)	0.90 (0.02)
Fall	− 0.14 (0.10)	1.01 (0.16)	1.51 (0.23)	0.89 (0.02)	0.94 (0.02)	0.93 (0.02)
Winter	− 0.32 (0.12)	1.09 (0.23)	1.64 (0.31)	0.84 (0.02)	0.87 (0.03)	0.86 (0.03)

Given in the table are the mean and standard deviation (in parenthesis) of the corresponding statistics for the years 1988–2015

On a seasonal scale, the generic model shows the best performance in spring and fall (R^2^ ≥ 0.94), followed by summer (R^2^ ≥ 0.92) and winter (R^2^ ≥ 0.87). There is a positive bias in summer, but a negative bias in all other seasons. The largest bias is found for winter with − 0.32 °C.

At JUN, the generic model shows a much lower predictability than at other sites (largest ME, MAE and lowest MIA, R^2^ and NSE, respectively; Table S4, Supplementary Material), indicating that applications of the generic model should be restricted to altitudes below about 1500 m.a.s.l or less.

### Accumulated growing degree-days and empirical correction

**Fig. 4:**
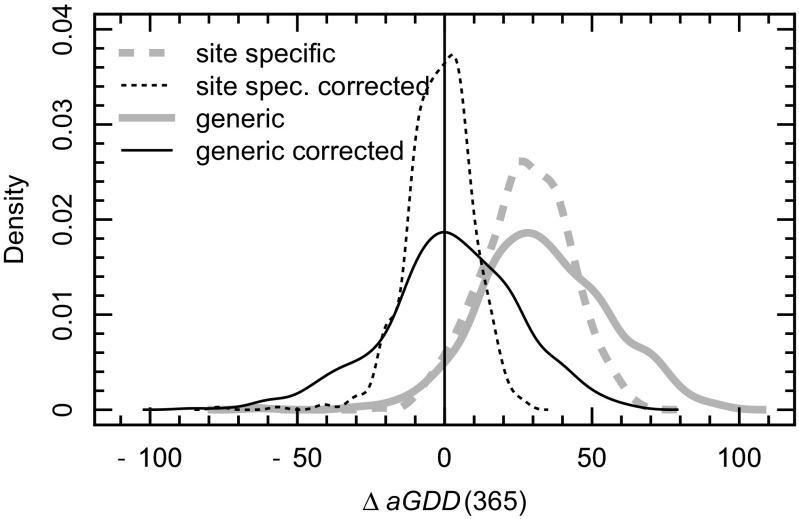
Probability distribution of the difference between simulated and actual aGDD at the end of the year (DOY 365) during the period 1988–2015 (excluding JUN; *n* = 532 site years)

Owing to the positive bias in *T* during the time of the day when *T* ≥ *T*_b_ (cf. area between dotted lines in Fig. 3), there is a systematic positive deviation of estimated versus actual aGDD at the end of each year (Fig. 4). This can be accommodated by applying an empirical correction factor (*f*_corr_). For the specific models, *f*_corr_ ranges between 0.958 and 0.989, whereas the value is 0.974 for the generic model.

### Inter-annual variability of accumulated growing degree-days

Yearly variations in aGDD and corresponding dates are simulated with high accuracy at all sites (Fig. S2, Supplementary Material). Because daily mean errors in simulated temperatures are also accumulated when computing aGDD, the model skill for the dates corresponding to aGDD ≤ 800 °C d is much better than for dates corresponding to aGDD > 800 °C d, with significant departures of estimated from actual dates appearing from time to time even at sites for which the model performance is otherwise excellent (e.g. BAS in 1993 or GUT in 2008).

Additional information concerning the model performance in simulating dates corresponding to aGDD of 200 and 800 °C d can be found in Tables S5 and S6 for the site-specific model and Tables S7 and S8 (Supplementary Material) for the generic model.

**Fig. 5:**
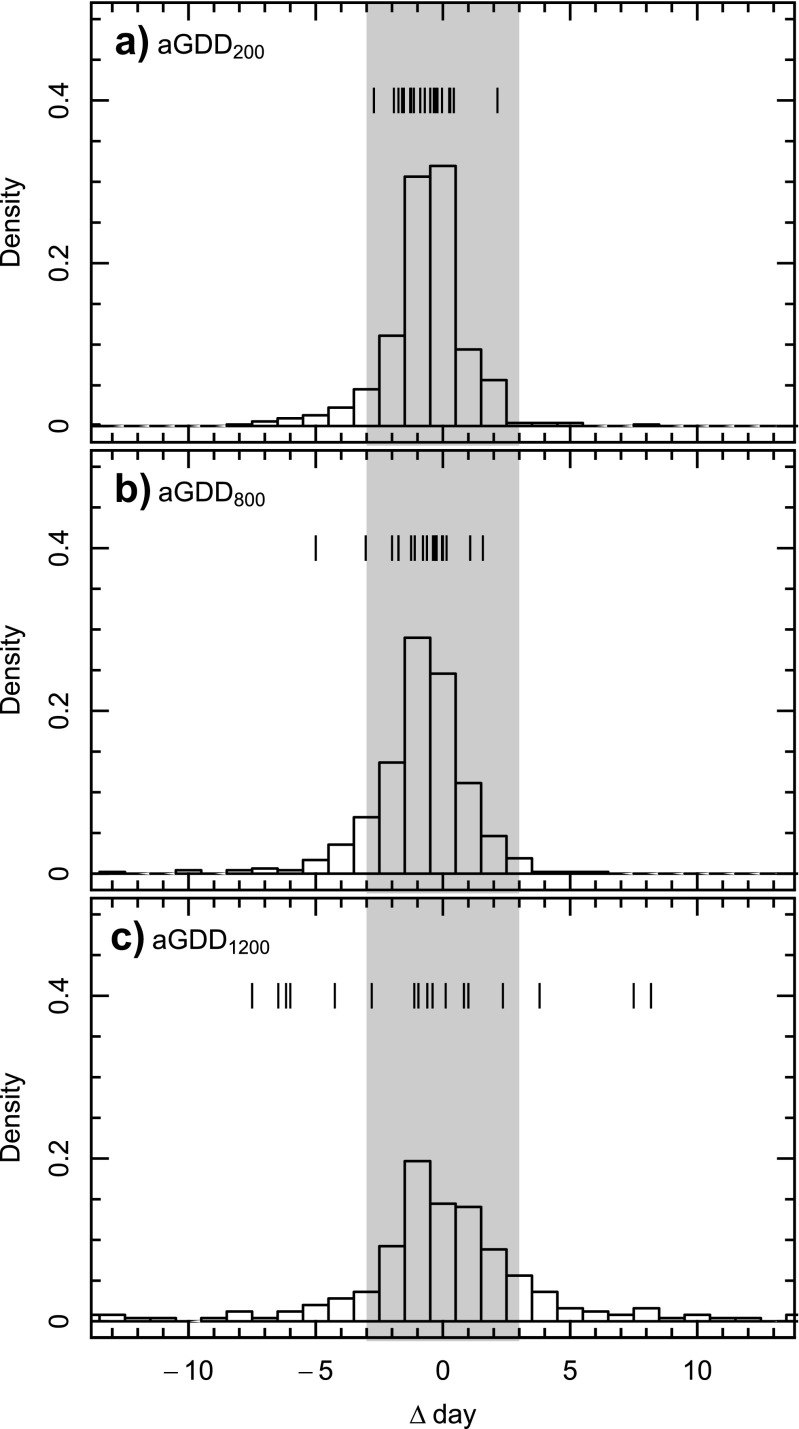
Probability distribution of the difference between simulated and actual DOY corresponding to aGDD = **a)** 200 °C d, **b)** 800 °C d and **c)** 1200 °C d. Vertical dashes show the mean differences at the individual sites (except JUN). The grey area highlights the range of differences bounded by ± 3 days

The probability distribution of the difference between simulated and actual dates corresponding to prescribed aGDD discloses a small tendency for the generic model to anticipate the actual dates (Fig. 5). For aGDD ≤ 800 °C d, most of the differences lie within a window of ± 3 days, indicating a high efficiency (Ef > 0.8). However, the efficiency drops below 0.5 for dates corresponding to aGDD = 1200 °C d (Table 3).

**Table 3:** Percentage of sites reaching accumulated growing degree-day (aGDD) values of 100, 200 and 1200 °C d (*T*_b_ = 10 °C) for model performance (Ef > 0.8 and Ef > 0.5) for the years 1988–2015

aGDD (°C d)	*N*	Years	Ef > 0.8 (%)	Ef > 0.5 (%)
200	19	532	100.0	100.0
800	18	476	88.9	94.4
1200	17	249	35.3	64.7

*N* denotes the number of sites reaching the aGDD value, years indicate the total number of years summed over all sites reaching the aGDD value

### Spatial application of the generic temperature model to calculate thermal heat sums

**Fig. 6:**
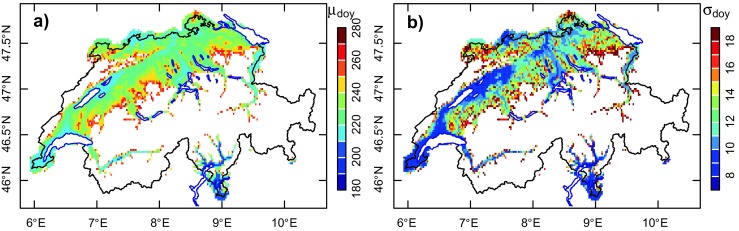
Spatial distribution of **a)** the mean DOY corresponding to 800 °C d and **b)** the corresponding inter-annual variability (standard deviation) for 1981–2015

To illustrate the potential for application of the generic model to the spatial analysis of plant and insect phenology, the distribution of the mean date of occurrence of 800 °C d (*T*_b_ = 10 °C) between 1981 and 2015, along with the associated inter-annual standard deviation, is displayed in Fig. 6. For this analysis, gridded data of daily ${T}_{\mathrm{n}}$ and *T*_x_ at 0.02° × 0.02° spatial resolution (approximately 2.2 × 2.2 km) were used.

In the complex topographic settings of Switzerland, mean phenological development is primarily a function of altitude, with aGDD = 800 °C d occurring in August on the plateau (DOY ∈ [213:243]), but during the second half of September at about 1000 m.a.s.l. The different thermal regimes to the north and south of the Alps are also nicely reflected in Fig. 6a, with earlier dates being predominant in Southern Switzerland.

For this particular value of aGDD, the inter-annual variability of the date of occurrence is large, the standard deviation being about 10 days on the plateau, but 18 days in the Jura Mountains and the Fore-Alps.

## Discussion

### Need for models and choice of the model

There are situations in which aGDD estimated from daily data are by no means worse than estimates obtained from hourly data. This is the case when the time interval of integration is short and the base temperature low. For instance, Purcell (2003) found that for warm-season crops, there was no significant difference in using hourly or daily data to assess the time needed to cumulate 200 °C d. In general, however, predictions of aGDD and corresponding dates based on hourly temperature data are superior to predictions based on daily data (Worner 1988; Gu 2016). Accordingly, various models have been proposed to simulate diurnal temperature variations from ${T}_{\mathrm{n}}$ and ${T}_{\mathrm{x}}$.

Of course, it is pertinent to ask whether such models are still needed. In fact, meteorological data are nowadays routinely sampled at frequencies higher than the daily. In practice, however, sub-daily scale temperature data are not always accessible. In particular, gridded data developed for or employed in agricultural and biometeorological investigations, such as WorldClim (Hijmans et al. 2005), CliMond (Kriticos et al. 2012), CHELSA (Karger et al. 2016a, b), various versions of the CRU (Climate Research Unit, University of East Anglia) data (e.g. Harris et al. 2014), the Global Climate Data Repository of the University of Delaware (Willmott and Robeson 1995), E-OBS (Haylock et al. 2008), as well as other continental (NRC 2017), regional (Daymet 2017) or national (Srivastava et al. 2009; Aalto et al. 2016) gridded data repositories, are only available at monthly or, at the best, daily time resolution. Similar considerations hold true concerning climate change scenarios.

For the present investigation, we opted for the model proposed by Parton and Logan (1981), not because we think that it is in itself superior, but rather because it provides a pragmatic workaround. In line with Eckersten (1986) and Eccel (2010b), the original model was modified for smoothed day-to-day transitions to prevent temperature jumps between days. It was also corrected to force the simulated temperature curve through *T*_n_ and ${T}_{\mathrm{x}}$.

### Model parameters

Another chief advantage of Parton and Logan’s (1981) model is that all parameters have a physical meaning, being either time shifts (*a* and *c*) related to the delayed effect of radiation on temperature or defining the exponential decay of temperature (*b*) caused by radiational cooling during night. This facilitates the model calibration since initial estimates of the parameter values are easily obtained from visual inspection of a few data. In addition, two of the parameters (*a* and *c*) are primarily determined by astronomical settings, implying that spatial variations of their values are modest, as long as the latitudinal extent of the area of interest is not too broad. Our results show this being indeed the case for Switzerland, confirming the conclusions drawn for other regions of the world (Reicosky et al. 1989; Cesaraccio et al. 2001). As a further simplification, they can be assumed as constant throughout the year, although in theory, one could consider letting their value vary depending on the season (Cesaraccio et al. 2001).

### Model performance: hourly temperatures

In our study, the generic model performed slightly better than in the original application discussed by Parton and Logan (1981). MAE and RMSD were in the range of 0.99 ± 0.07 and 1.5 ± 0.11 °C at the calibration sites and 1.05 ± 0.16 and 1.60 ± 0.23 °C at the validation sites, respectively, compared to nominal values of 2.35 and 3.14 °C as found by Parton and Logan (1981). Applying the original model as well, Reicosky et al. (1989) found MAE and RMSD of 1.67 and 2.08 °C for randomly selected days. Cesaraccio et al. (2001) found RMSD of 2.93 °C for five sites in California during the period 1996–1999. Concerning the model performance for individual seasons, Cesaraccio et al. (2001) and Purcell (2003) found best predictability (R^2^) for summer temperatures. In our study, the best performance was found for spring and fall temperatures.

Both site-specific and the generic models showed a tendency to overestimate temperatures in the late afternoon but underestimate temperature in the early morning (Fig. 3 and Fig. S1, Supplementary Material). We argued that this is due to the choice of sinusoidal variations to model day-time temperatures and exponential decay to model night-time temperatures. More complex formulations have been proposed to improve the performance of this type of model (Wilson and Barnett 1983; Eckersten 1986; Roltsch et al. 1999; Cesaraccio et al. 2001; Eccel 2010a), but these come at the expense of a larger number of parameters that need to be calibrated.

Even more refined models cannot account for departures from the expected behaviour caused by synoptic disturbances (Purcell 2003) or induced by the specificities of the local topography (Cesaraccio et al. 2001). A problem often encountered in such circumstances is that *T*_n_ and *T*_x_ do not necessarily occur around sunrise and in the early afternoon, respectively, as usually assumed by the models (Linvill 1990). In a study carried out in the Trento region, Italy, Eccel (2010b) found that in 20% of the days, *T*_n_ occurred after midday during the years 1983–2009. The analysis of the timing of ${T}_{\mathrm{n}}$ and ${T}_{\mathrm{x}}$ in the data used for our model calibration showed that on 27% of the days, ${T}_{\mathrm{n}}$ does occur in the afternoon. Similarly, on 13% of the days, ${T}_{\mathrm{x}}$ was found to occur before noon or after sunset. Unfortunately, daily temperature records do not report the time of occurrence of ${T}_{\mathrm{n}}$ and ${T}_{\mathrm{x}}$, implying that the problem cannot be easily resolved. Statistical or dynamic downscaling (e.g. Calanca et al. 2009; Hirschi et al. 2012a, b) could be considered to circumvent this problem, but they rely even more heavily on the availability of hourly temperature data than the current approach.

### Model performance: accumulated growing degree-days

Owing to the slight but systematic overestimation of hourly temperatures by the model, estimated aGDD showed a positive bias at the end of the year. Even if not shown, this type of error is known to depend on the choice of the base temperature (*T*_b_). As pointed out by Worner (1988), a lower *T*_b_ would increase the number of hours included on a given day in the computation of aGDD, resulting (in our case) in a larger compensation of the positive bias during the afternoon by the negative bias of the early morning hours. Notwithstanding, the introduction of empirical correction factors (Allen 1976; Pruess 1983) can be recommended. Our results showed a significant improvement after the application of site-specific correction factors and modest improvements after application of a generic correction factor derived from a relatively small number of sites. In both cases, the model efficiency in predicting dates corresponding to prescribed aGDD value was by and large satisfactory (0.8 for aGDD ≤ 800 °C d, 0.5 for aGDD above this threshold; cf. Table 3).

## Conclusions

The ability to reliably predict phenological dates of crops and insects is of paramount importance for informing agricultural management. Many decision support systems developed for this purpose adopt accumulated growing degree-days as a basis for estimating phenological stages and require hourly temperature data on input. Despite increasing availability of temperature data at sub-daily timescales, there are still many situations in which hourly temperatures need to be derived from daily aggregated data. Models for predicting diurnal temperature variations are essential in this context. In this work, we showed that even a generic calibration of this type of model can deliver reliable inputs for assessing crop and insect phenology in space and time, opening opportunities for extending the range of application of current decision support systems.

## Electronic supplementary material

ESM 1 (PDF 1532 kb)
