# Supplementary material for: Generic calibration of a simple model of diurnal temperature variations for spatial analysis of accumulated degree-days
Source: Int J Biometeorol. 2017 Dec 7;62(4):621–30. doi: 10.1007/s00484-017-1471-5 (PMC5874280; doi:10.1007/s00484-017-1471-5)
Supplement: Supplementary file 1 — (PDF 1532 kb) [file 484_2017_1471_MOESM1_ESM.pdf]

Supplementary Material

Felber R[1)], Stoeckli S, Calanca P. (20xx), Generic calibration of a simple model of diurnal temperature variations for spatial analysis of accumulated degree-days. Int J Biometeorol

[1)]Agroscope, Research Division Agroecology and Environment, Climate and Air Pollution Group, Zurich, Switzerland; raphael.felber@agroscope.admin.ch

Table S1 Location (Lon/Lat) and altitude (Height) of selected meteorological stations in Switzerland and available years of temperature data (Years) used in this study. Column Cal/val indicates which sites were used for model calibration (cal) and validation (val). The years 1983, 1984, 1986–1990, 1992–1998, 2000–2002, 2004–2007, 2009, 2011–2013 were used for model calibration (if available), whereas the remaining years were used for model validation

**Station name**	**Site**	**Lon**	**Lat**	**Height** [m a.s.l.]	**Years**	**Cal/val**
Aadorf/Tänikon	TAE	8°54'	47°29'	539	1988–2015	val
Basel/Binningen	BAS	7°35'	47°32'	316	1981–2015	cal/val
Bern/Zollikofen	BER	7°28'	46°59'	552	1981–2015	cal/val
Buchs/Aarau	BUS	8°05'	47°23'	386	1985–2015	cal/val
Bullet/La Frétaz	FRE	6°35'	46°50'	1205	1988–2015	val
Chur	CHU	9°32'	46°52'	556	1981–2015	cal/val
Davos	DAV	9°51'	46°49'	1594	1988–2015	val
Güttingen	GUT	9°17'	47°36'	440	1981–2015	cal/val
Interlaken	INT	7°52'	46°40'	577	1988–2015	val
Jungfrau	JUN	7°59'	46°33'	3580	1988–2015	val
Lugano	LUG	8°58'	46°00'	273	1988–2015	val
Magadino/Cadenazzo	MAG	8°56'	46°10'	203	1981–2015	cal/val
Nyon/Changins	CGI	6°14'	46°24'	455	1981–2015	cal/val
Pully	PUY	6°40'	46°31'	455	1988–2015	val
Sion	SIO	7°20'	46°13'	482	1981–2015	cal/val
St.Gallen	STG	9°24'	47°26'	775	1982–2015	cal/val
Visp	VIS	7°51'	46°18'	639	1988–2015	val
Wädenswil	WAE	8°41'	47°13'	485	1981–2015	cal/val
Wynau	WYN	7°47'	47°15'	422	1988–2015	val
Zürich/Affoltern	REH	8°31'	47°26'	443	1988–2015	val

Table S2 Optimized model parameters (*a, b,* and *c*) for the calibration (top) and validation sites (bottom). Indicated in the table are the number of 'clear-sky' days (n) and the modified index of agreement (MIA; Legates and McCabe 1999). The total number of years of the calibration sites is 25 years, whereas the total number of years of the validation sites is 21 years

Site	*a*	*b*	*c*	n	MIA
**Calibration sites**					
BAS	2.83	3.18	0.83	1376	0.96
BER	2.80	3.44	0.62	1326	0.96
BUS	2.86	3.56	0.75	790[a)]	0.95
CGI	2.81	2.52	0.38	1314	0.94
CHU	2.39	3.29	1.02	989	0.95
GUT	2.91	3.31	0.83	982	0.93
MAG	2.32	2.63	0.39	1185	0.94
SIO	2.60	3.15	0.83	1809	0.95
STG	3.13	2.95	1.17	1175	0.94
WAE	3.24	3.55	1.14	1144	0.95
**Validation sites**					
DAV	1.91	3.26	0.85	1720	0.96
FRE	2.31	2.88	0.30	933	0.94
INT	2.55	3.31	1.40	1370	0.95
JUN	12.26	1.63	-2.41	1319	0.92
LUG	2.96	2.98	1.21	830	0.95
PUY	5.85	3.16	0.53	1600	0.94
REH	2.88	3.45	0.59	1194	0.95
TAE	2.50	3.61	0.42	1086	0.94
VIS	2.52	3.12	0.69	1329	0.94
WYN	2.55	3.46	1.00	1077	0.95

[a)] Measured data starts at year 1985, thus for the optimization two years less could be used

Table S3 Performance statistics (and their mean and standard deviation across sites) of the *site specific models*: mean error (ME), mean absolute error (MAE), root mean square deviation (RMSD), modified index of agreement (MIA), coefficient of determination (R^2), Nash-Sutcliffe efficiency (NSE). The last row indicates the mean and standard deviation (SD) of all sites

**Site**	**ME** [°C]	**MAE** [°C]	**RMSD** [°C]	**MIA**	$\mathbf{R^2}$	**NSE**
**Calibration sites**[1)]						
BAS	−0.06	0.91	1.43	0.93	0.97	0.97
BER	−0.11	0.94	1.46	0.93	0.97	0.97
BUS	−0.11	0.98	1.50	0.93	0.97	0.97
CGI	0.04	0.91	1.34	0.93	0.97	0.97
CHU	−0.09	1.03	1.58	0.93	0.97	0.96
GUT	−0.05	1.06	1.56	0.92	0.97	0.96
MAG	−0.04	0.92	1.39	0.94	0.97	0.97
SIO	0.02	0.98	1.44	0.93	0.97	0.97
STG	−0.08	1.09	1.72	0.92	0.96	0.95
WAE	−0.07	0.94	1.45	0.93	0.97	0.97
Mean (SD)	−0.06 (0.05)	0.98 (0.06)	1.50 (0.11)	0.93 (0.02)	0.97 (0.003)	0.97 (0.01)
**Validation sites**[2)]						
DAV	−0.28	1.02	1.56	0.92	0.97	0.96
FRE	−0.04	1.01	1.52	0.92	0.96	0.96
INT	−0.10	0.95	1.44	0.93	0.97	0.97
JUN	−0.26	1.22	1.80	0.89	0.93	0.92
LUG	−0.01	0.74	1.16	0.94	0.98	0.98
PUY	−0.06	0.87	1.32	0.93	0.97	0.97
REH	−0.13	1.00	1.51	0.93	0.97	0.96
TAE	−0.26	1.18	1.78	0.91	0.96	0.95
VIS	−0.13	1.14	1.69	0.92	0.97	0.96
WYN	−0.13	0.97	1.46	0.93	0.97	0.97
Mean (SD)	−0.14 (0.10)	1.00 (0.14)	1.50 (0.20)	0.92 (0.01)	0.96 (0.01)	0.96 (0.02)

[1)] Data for the calibrations sites shown here comprise only the validation years, i.e. 1981, 1982, 1985, 1991, 1999, 2003, 2008, 2010, 2014, 2015, except BUS for which the years 1981 and 1982 are missing and STG for which the year 1981 is missing.

[2)] Data for the validation sites comprise the years 1988–2015.

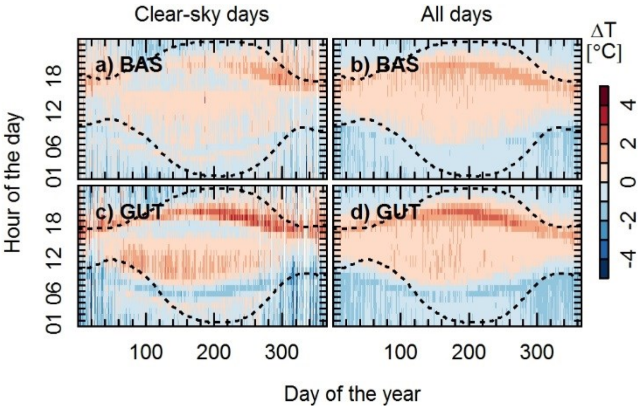

Fig. S1 Mean for 1981–2015 of the difference between simulated and actual temperatures as a function of the time of the day (y-axis) and day of the year (x-axis), at BAS (upper row) and GUT (lower row). Panels on the left present results for clear-sky days only, whereas panels on the right show the results obtained when using all days. Reddish/blueish colours indicate a positive/negative bias. The dotted lines delimit, on the insight, the time of the day when T is in excess of T_b = 10°C

Table S4 Performance statistics (and their mean and standard deviation across sites) of the *generic model*: mean error (ME), mean absolute error (MAE), root mean square deviation (RMSD), modified index of agreement (MIA), coefficient of determination (R^2), Nash-Sutcliffe efficiency (NSE). The last row indicates the mean and standard deviation (SD) of all sites

**Site**	**ME** [°C]	**MAE** [°C]	**RMSD** [°C]	**MIA**	**R^2**	**NSE**
**Calibration sites**[1)]						
BAS	−0.06	0.91	1.42	0.93	0.97	0.97
BER	−0.09	0.93	1.44	0.93	0.97	0.97
BUS	−0.05	0.96	1.46	0.93	0.97	0.97
CGI	−0.17	0.93	1.38	0.93	0.97	0.97
CHU	0.09	1.07	1.63	0.92	0.96	0.96
GUT	−0.03	1.05	1.54	0.92	0.97	0.96
MAG	−0.17	0.99	1.47	0.93	0.97	0.97
SIO	0.06	0.99	1.46	0.93	0.97	0.97
STG	−0.09	1.11	1.74	0.92	0.96	0.95
WAE	0.01	0.93	1.43	0.93	0.97	0.97
Mean (SD)	−0.05 (0.09)	0.99 (0.07)	1.50 (0.11)	0.93 (0.01)	0.97 (0.004)	0.97 (0.01)
**Validation sites**[2)]						
DAV	−0.01	1.15	1.70	0.91	0.96	0.95
FRE	−0.10	1.04	1.56	0.91	0.96	0.95
INT	0.12	0.99	1.48	0.93	0.97	0.97
JUN	−0.15	1.32	1.98	0.88	0.91	0.91
LUG	0.01	0.77	1.21	0.94	0.98	0.97
PUY	−0.03	0.87	1.30	0.93	0.97	0.97
REH	−0.12	0.98	1.48	0.93	0.97	0.97
TAE	−0.21	1.19	1.77	0.91	0.96	0.95
VIS	−0.10	1.16	1.71	0.92	0.97	0.96
WYN	0.02	0.98	1.47	0.93	0.97	0.97
Mean (SD)	−0.06 (0.10)	1.05 (0.16)	1.60 (0.23)	0.92 (0.02)	0.96 (0.02)	0.96 (0.02)

1) Data for the calibrations sites shown here comprise only the validation years, i.e. 1981, 1982, 1985, 1991, 1999, 2003, 2008, 2010, 2014, 2015, except BUS for which the years 1981 and 1982 are missing and STG for which the year 1981 is missing.

2) Data for the validation sites comprise the years 1988–2015.

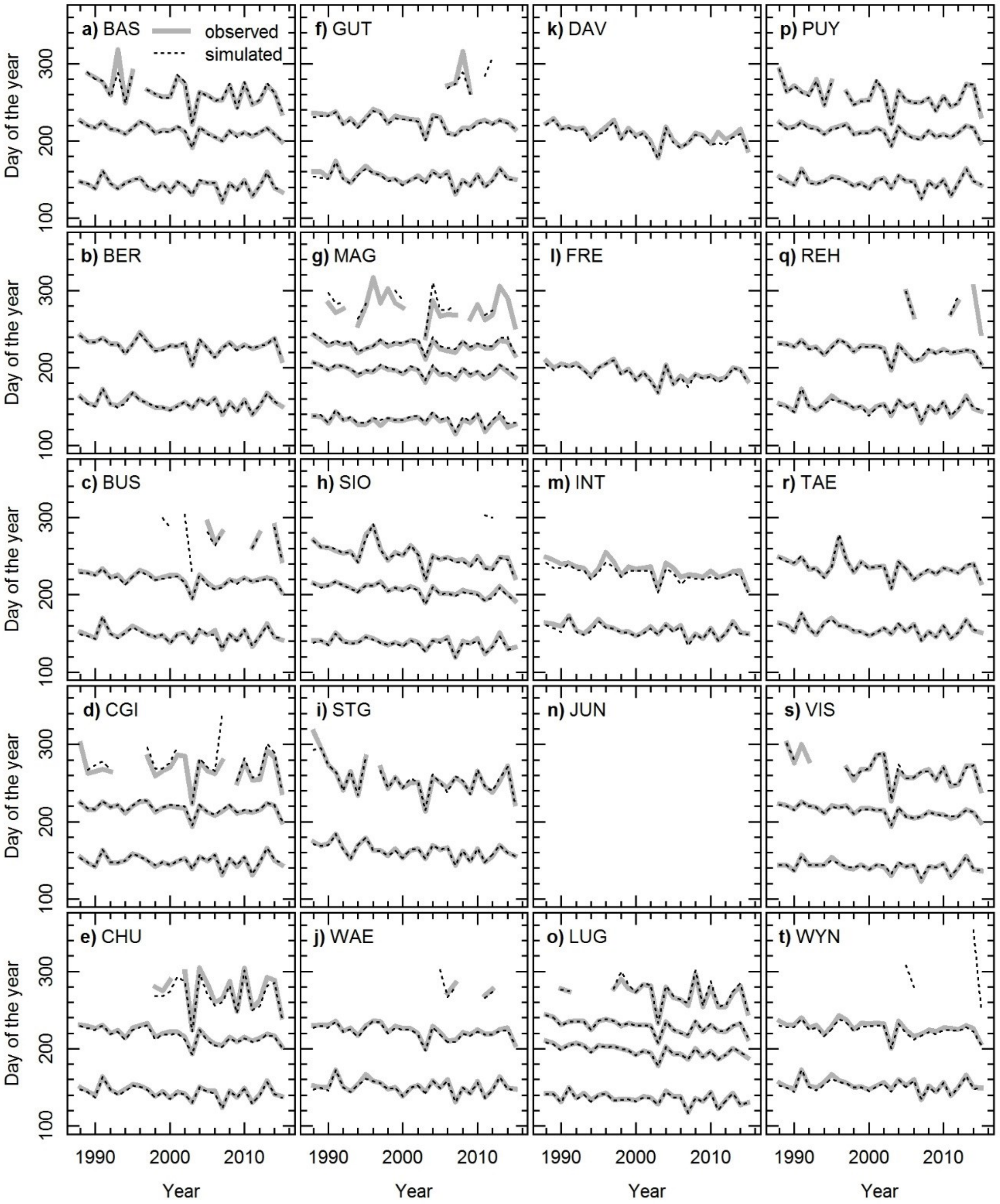

Fig. S2 Simulated and observed day of the year when accumulated growing degree-days of 200°C d, 800°C d, 1200°C d, and 1600°C d (T_b = 10°C) are attained (lines from bottom to top in each panel). Plotted are the results of the generic model with empirical correction for the calibration sites **(a–j)** and the validation sites **(k–t)**

Table S5 Performance of the *site-specific models* in predicting accumulated degree-days. Results are for 200°C d (T_b = 10°C), as evaluated from observed (Obs), and simulated hourly temperature data, without (Sim) and with (Corr) empirical correction (f_{corr}). Left-hand side: Mean DOY and Standard Deviation (SD) for 1988-2015. Right-hand side: results of the Wilcoxon-Mann-Whitney test (WMW), the Kolmogorov-Smirnoff test (KS), and the model efficiency (Ef) for the comparison between the corresponding distributions. In all cases, n = 28

		**Mean DOY**			**SD**			**Simulated**			**Corrected**		
**Site**	f_{corr}	**Obs**	**Sim**	**Corr**	**Obs**	**Sim**	**Corr**	**WMW**	**KS**	**Ef**	**WMW**	**KS**	**Ef**
BAS	0.975	142	142	143	8.7	8.5	8.4	n.s.	n.s.	100.0	n.s.	n.s.	100.0
BER	0.970	154	153	154	8.1	7.9	7.8	n.s.	n.s.	89.3	n.s.	n.s.	96.4
BUS	0.965	148	147	148	8.6	8.0	8.4	n.s.	n.s.	92.9	n.s.	n.s.	100.0
CGI	0.963	149	147	148	8.2	7.7	7.7	n.s.	n.s.	89.3	n.s.	n.s.	100.0
CHU	0.985	144	143	144	8.5	8.2	8.2	n.s.	n.s.	96.4	n.s.	n.s.	96.4
GUT	0.958	153	150	152	8.8	8.3	8.4	n.s.	n.s.	71.4	n.s.	n.s.	92.9
MAG	0.989	132	132	133	6.7	6.2	6.1	n.s.	n.s.	96.4	n.s.	n.s.	96.4
SIO	0.977	138	136	137	7.3	6.5	6.6	n.s.	n.s.	96.4	n.s.	n.s.	96.4
STG	0.985	163	161	162	9.5	9.5	9.4	n.s.	n.s.	92.9	n.s.	n.s.	96.4
WAE	0.971	151	150	151	9.0	8.8	8.8	n.s.	n.s.	89.3	n.s.	n.s.	96.4

Table S6 Performance of the *site-specific models* in predicting accumulated degree-days. Results are for 800°C d (T_b = 10°C), as evaluated from observed (Obs), and simulated hourly temperature data, without (Sim) and with (Corr) empirical correction (f_{corr}). Left-hand side: Mean DOY and Standard Deviation (SD) for 1988-2015. Right-hand side: results of the Wilcoxon-Mann-Whitney test (WMW), the Kolmogorov-Smirnoff test (KS), and the model efficiency (Ef) for the comparison between the corresponding distributions. Except at STG (n = 27), n = 28

		**Mean DOY**			**SD**			**Simulated**			**Corrected**		
**Site**	f_{corr}	**Obs**	**Sim**	**Corr**	**Obs**	**Sim**	**Corr**	**WMW**	**KS**	**Ef**	**WMW**	**KS**	**Ef**
BAS	0.975	213	210	212	8.0	7.9	7.9	n.s.	n.s.	89.3	n.s.	n.s.	100.0
BER	0.970	229	225	228	9.2	8.4	9.0	n.s.	n.s.	64.3	n.s.	n.s.	100.0
BUS	0.965	220	217	220	8.6	8.2	8.2	n.s.	n.s.	67.9	n.s.	n.s.	100.0
CGI	0.963	216	213	216	7.5	7.2	7.3	<0.05	n.s.	57.1	n.s.	n.s.	100.0
CHU	0.985	218	216	217	9.7	9.5	9.5	n.s.	n.s.	100.0	n.s.	n.s.	100.0
GUT	0.958	226	222	225	9.4	8.9	9.3	n.s.	n.s.	35.7	n.s.	n.s.	96.4
MAG	0.989	196	195	195	6.2	5.9	5.9	n.s.	n.s.	100.0	n.s.	n.s.	100.0
SIO	0.977	206	204	206	7.3	7.6	7.5	n.s.	n.s.	89.3	n.s.	n.s.	100.0
STG	0.985	255	249	252	21.4	16.8	18.7	n.s.	n.s.	40.7	n.s.	n.s.	70.4
WAE	0.971	223	220	223	9.0	8.7	8.8	n.s.	n.s.	71.4	n.s.	n.s.	100.0

Table S7 Performance of the *generic model* in predicting accumulated degree-days. Results are for 200°C d (T_b = 10°C), as evaluated from observed (Obs), and simulated hourly temperature data, without (Sim) and with (Corr) empirical correction (f_{corr}). Left-hand side: Mean DOY and Standard Deviation (SD) for 1988-2015. Right-hand side: results of the Wilcoxon-Mann-Whitney test (WMW), the Kolmogorov-Smirnoff test (KS), and the model efficiency (Ef) for the comparison between the corresponding distributions. Except at JUN (n = 0), n = 28

		**Mean DOY**			**SD**			**Simulated**			**Corrected**		
**Site**	f_{corr}	**Obs**	**Sim**	**Corr**	**Obs**	**Sim**	**Corr**	**WMW**	**KS**	**Ef**	**WMW**	**KS**	**Ef**
BAS	0.974	142	142	143	8.7	8.5	8.4	n.s.	n.s.	100.0	n.s.	n.s.	100.0
BER	0.974	154	153	154	8.1	8.0	7.8	n.s.	n.s.	89.3	n.s.	n.s.	96.4
BUS	0.974	148	147	148	8.6	8.0	8.4	n.s.	n.s.	92.9	n.s.	n.s.	96.4
CGI	0.974	149	148	149	8.2	7.6	7.8	n.s.	n.s.	100.0	n.s.	n.s.	100.0
CHU	0.974	144	142	143	8.5	8.3	8.3	n.s.	n.s.	82.1	n.s.	n.s.	96.4
GUT	0.974	153	150	152	8.8	8.3	8.4	n.s.	n.s.	71.4	n.s.	n.s.	89.3
MAG	0.974	132	133	134	6.7	6.2	6.3	n.s.	n.s.	92.9	n.s.	n.s.	85.7
SIO	0.974	138	136	137	7.3	6.4	6.6	n.s.	n.s.	82.1	n.s.	n.s.	96.4
STG	0.974	163	161	162	9.5	9.5	9.3	n.s.	n.s.	92.9	n.s.	n.s.	100.0
WAE	0.974	151	149	150	9.0	8.8	8.6	n.s.	n.s.	85.7	n.s.	n.s.	89.3
DAV	0.974	208	204	206	11.7	11.5	11.6	n.s.	n.s.	60.7	n.s.	n.s.	82.1
FRE	0.974	194	191	192	10.3	10.5	10.4	n.s.	n.s.	75.0	n.s.	n.s.	89.3
INT	0.974	155	152	153	8.1	7.7	7.9	n.s.	n.s.	75.0	n.s.	n.s.	85.7
JUN	0.974	NA	NA	NA	NA	NA	NA	NA	NA	NA	NA	NA	NA
LUG	0.974	136	134	135	7.2	6.9	7.0	n.s.	n.s.	96.4	n.s.	n.s.	100.0
PUY	0.974	147	145	146	8.4	8.2	8.2	n.s.	n.s.	92.9	n.s.	n.s.	100.0
REH	0.974	150	149	150	9.0	8.4	8.4	n.s.	n.s.	85.7	n.s.	n.s.	92.9
TAE	0.974	156	155	156	8.3	8.0	8.0	n.s.	n.s.	96.4	n.s.	n.s.	100.0
VIS	0.974	142	141	142	7.3	6.5	6.4	n.s.	n.s.	96.4	n.s.	n.s.	92.9
WYN	0.974	153	151	151	8.1	7.9	7.8	n.s.	n.s.	85.7	n.s.	n.s.	85.7

Table S8 Performance of the *generic model* in predicting accumulated degree-days. Results are for 800°C d (T_b = 10°C), as evaluated from observed (Obs), and simulated hourly temperature data, without (Sim) and with (Corr) empirical correction (f_{corr}). Left-hand side: Mean DOY and Standard Deviation (SD) for 1988-2015. Right-hand side: results of the Wilcoxon-Mann-Whitney test (WMW), the Kolmogorov-Smirnoff test (KS), and the model efficiency (Ef) for the comparison between the corresponding distributions. Except at JUN and DAV (n = 0), FRE (n = 1) and STG (n = 27), n = 28

		**Mean DOY**			**SD**			**Simulated**			**Corrected**		
**Site**	f_{corr}	**Obs**	**Sim**	**Corr**	**Obs**	**Sim**	**Corr**	**WMW**	**KS**	**Ef**	**WMW**	**KS**	**Ef**
BAS	0.974	213	210	213	8.0	7.9	8.0	n.s.	n.s.	89.3	n.s.	n.s.	100.0
BER	0.974	229	226	228	9.2	8.5	9.0	n.s.	n.s.	71.4	n.s.	n.s.	100.0
BUS	0.974	220	217	219	8.6	8.2	8.3	n.s.	n.s.	53.6	n.s.	n.s.	100.0
CGI	0.974	216	215	218	7.5	7.3	7.5	n.s.	n.s.	100.0	n.s.	n.s.	100.0
CHU	0.974	218	214	216	9.7	9.2	9.5	n.s.	n.s.	32.1	n.s.	n.s.	96.4
GUT	0.974	226	222	224	9.4	9.0	9.1	n.s.	n.s.	32.1	n.s.	n.s.	96.4
MAG	0.974	196	195	197	6.2	5.9	5.7	n.s.	n.s.	100.0	n.s.	n.s.	100.0
SIO	0.974	206	203	205	7.3	7.4	7.5	n.s.	n.s.	85.7	n.s.	n.s.	96.4
STG	0.974	255	249	255	21.4	16.8	19.1	n.s.	n.s.	40.7	n.s.	n.s.	81.5
WAE	0.974	223	219	221	9.0	8.7	8.8	n.s.	n.s.	42.9	n.s.	n.s.	96.4
DAV	0.974	NA	NA	NA	NA	NA	NA	NA	NA	NA	NA	NA	NA
FRE	0.974	261	247	261	NA	NA	NA	NA	NA	0.0	NA	NA	100.0
INT	0.974	232	224	227	10.9	9.2	9.6	<0.05	n.s.	3.6	n.s.	n.s.	32.1
JUN	0.974	NA	NA	NA	NA	NA	NA	NA	NA	NA	NA	NA	NA
LUG	0.974	197	195	197	7.5	7.2	7.3	n.s.	n.s.	100.0	n.s.	n.s.	100.0
PUY	0.974	213	210	212	7.7	7.5	7.6	n.s.	n.s.	85.7	n.s.	n.s.	100.0
REH	0.974	223	220	223	9.0	8.7	8.9	n.s.	n.s.	85.7	n.s.	n.s.	100.0
TAE	0.974	235	232	235	12.2	10.9	12.6	n.s.	n.s.	85.7	n.s.	n.s.	96.4
VIS	0.974	213	211	213	7.5	7.3	7.1	n.s.	n.s.	82.1	n.s.	n.s.	100.0
WYN	0.974	227	222	224	9.1	8.6	8.6	<0.05	n.s.	14.3	n.s.	n.s.	67.9

References

Legates DR, McCabe GJ (1999) Evaluating the use of “goodness-of-fit” Measures in hydrologic and hydroclimatic model validation. Water Resour Res 35:233–241. doi: 10.1029/1998WR900018
